# Supplementary material for: Are carbon emissions trading and green financial instruments synergistic? -Comprehensive quantitative research based on content analysis
Source: PLoS One. 2024 Mar 7;19(3):e0298601. doi: 10.1371/journal.pone.0298601 (PMC10919601; doi:10.1371/journal.pone.0298601)
Supplement: S1 Data — (ZIP) [file pone.0298601.s001.zip › Data/地级市Z_F工作报告.zip!/╘╞─╧/┴┘▓╫╩╨/└┤╫╘╣1⁄2╓┌║┼í╛┬φ┐╦╩2╛▌═°í┐_2015.docx]

各位代表：

　　现在，我代表市人民政府，向大会报告工作，请予审议，并请政协委员提出意见。

　　一、2014年工作回顾

　　2014年是本届政府履职的第一年。面对经济下行压力加大、自然灾害频繁发生等严峻形势，市人民政府在省委、省政府和市委的坚强领导下，全面贯彻党的十八大、十八届三中、四中全会精神和省委九届八次、九次全会精神，以邓小平理论、“三个代表”重要思想、科学发展观为指导，深入学习贯彻习近平总书记系列重要讲话精神，按照市委三届六次、七次全会的部署，自觉接受市人大及其常委会的法律监督、工作监督和市政协的民主监督，团结和依靠全市各族人民，以认真开展党的群众路线教育实践活动凝聚改革发展正能量，深入实施 “生态立市，绿色崛起”发展战略，坚持“三稳三进”，全面深化改革，以“世界佤乡、天下茶尊，恒春之都、大美临沧”为城市品牌定位，以“构筑生态高地，成就大美临沧”为目标，以边境一线、澜沧江一线、南汀河一线，边境经济合作区、临沧主城区、滇中产业聚集区临沧产业园，森林化、产业化、城镇化的 “三线三区三化”发展为重点，以临沧边境经济合作区建设为突破，抓项目、破瓶颈、彰特色、促跨越，加快推进总部经济、飞地经济，着力打好县域经济、园区经济、民营经济三大战役，加快“10大产业、10大园区、10大基础设施”建设，着重抓好42项重要指标、20项重点工作、20个重大项目、21户重点工业企业、10件惠民实事和“四个一百”上亿元重点项目，全力建设天地人高度和谐的大美临沧，较好的完成了市人大三届一次会议确定的各项目标任务。实现生产总值465.1亿元，增长11.2%；规模以上固定资产投资564.8亿元，增长35.4%；一般公共预算收入37.3亿元，增长1.1%；城镇常住居民人均可支配收入19526元，增长9.1%；农村常住居民人均可支配收入7199元，增长14.2%；社会消费品零售总额132.2亿元，增长13.7%；外贸进出口总额42.6亿元，增长20.6%；人口自然增长率6‰；城镇登记失业率3.63%；居民消费价格总水平涨幅2.6%；预计单位生产总值能耗下降0.8%。全市呈现出经济发展、社会进步、民生改善、文化繁荣、民族团结、边疆稳定的良好局面。

　　（一）农业农村经济持续发展

　　特色农业提质增效。坚持生态化、品牌化，加快发展农业产业。全面落实粮食行政首长负责制，粮食总产量突破百万吨大关，达102万吨。全市新增澳洲坚果、咖啡、竹木等产业化基地172.9万亩，总面积达1974.8万亩。临沧被授予“中国精品咖啡豆示范区”称号。凤庆、耿马、沧源三个省级高原特色农业示范县建设稳步推进，在建和初步建成现代农业庄园42个；临翔至双江5个现代农业产业园、南汀河流域10个生态农业产业园建设取得新成效。预计畜牧业和渔业产值分别增长5.2%和11.3%，农业增加值142.7亿元，增长6.5%。

　　“三万三带”工程顺利实施。大力推广“核桃+魔芋”、“林果+魔芋”等模式，累计建成“万元山”80万亩、 “万元田”15万亩，新培育收入达2万元以上“万元人”10.2万人。南汀河流域“三带”建设加快推进，建成苗木产业带基地109.3万亩、生态景观带林荫大道178.8公里，启动5个林产品交易中心建设。

　　农村经济发展活力不断增强。深化农村综合改革，农垦、供销社、水利等管理体制改革扎实推进，农村市场主体培育、农村土地流转、农村基础设施建设、农村信息和金融服务等方面取得新成绩。不断创新农业经营体制机制，新增农业产业化组织102个，新增农业龙头企业14户。农业产业化龙头企业实现增加值42亿元，增长14.2%。

　　（二）工业转型升级加快

　　工业发展后劲增强。坚持绿色化、园区化，加快实施工业重大项目。企业项目申报难、土地落实难、银行贷款难等问题得到有效解决。17个项目前期工作稳步推进，167个项目顺利开工，143个续建项目加快推进，185个项目顺利竣工，完成工业规模以上固定资产投资147.3亿元，增长31.8%。全社会用电量21.3亿度，增长18%。支持工业创新能力建设，绿色农产品加工水平全面提升，工业清洁生产和节能降耗取得新成效。推进工业化与信息化融合发展，物联网技术应用加快推广。

　　工业园区建设加快。坚持工业集约集群集聚发展，全面完成工业园区总体规划，滇中产业新区临沧产业园规划加快推进，临沧工业园区和县域工业园区加快建设。全市工业园区实施175个项目，完成工业固定资产投资100亿元；新增入园企业42户，累计入园企业达334户，实现工业增加值60亿元。

　　工业企业发展壮大。认真落实加快工业发展政策措施，严格执行挂钩服务企业制度，企业的煤电油运等要素保障不断增强，产能充分释放。新增规模以上工业企业30户。大力实施中小企业成长工程、“两个10万元”微型企业培育工程。全市完成规模以上工业增加值116.3亿元，增长14.4%。民营经济创业活力明显增强，完成民营经济增加值200.1亿元，增长20.3%。

　　（三）第三产业加快发展

　　生态文化旅游产业发展壮大。坚持特色化、高端化，推进旅游、生态、文化、现代服务业“四位一体”融合发展。沧源国际旅游度假区建设加快，临沧茶马古镇、沧源摸你黑狂欢城等一批旅游品牌建设取得新进展，勐来、鲁史等一批特色旅游小镇，古墨、翁丁、冰岛等一批避暑避寒“恒春小镇”，腾龙、芒团等一批特色旅游村建设取得新成效。接待国内外旅游者601万人次，增长30.2%；实现旅游总收入42.7亿元，增长38.4%。

　　美誉度明显提升。成功举办第二届亚洲微电影艺术节和“金海棠”奖颁奖活动，持续办好“摸你黑”、“澜沧江啤酒狂欢节”、“永德芒果节”等活动。“全国卫视进临沧”、“大美临沧·天下茶仓—中国书画名家临沧茶山行”、“中华诗词名家临沧行”、“大美临沧”摄影美术作品展等活动扩大了临沧的影响。临沧获得“2014年中国避暑旅游城市”、“中国最美生态旅游示范市”、“2014中国最具茶文化魅力城市品牌”。沧源获得“中国最美生态旅游示范县”、“中国最美休闲度假旅游名县”称号。翁丁、冰岛入围“2014年中国十佳避暑小镇”。沧源、双江入选“中国深呼吸小城100佳”。

　　服务业加快发展。大型批发市场建设、大型商贸流通企业培育、乡村流通工程加快推进，连锁经营、特许经营和物流配送加快发展。新增限额以上批零住餐企业21户，累计达146户，限额以上零售额占社会消费品零售总额比重由29.9%提高到47.9%。电子商务启动发展，中国网库“云南生物产业电子商务基地”一期15个单品平台建成运营。第三产业增加值123.6亿元，增长10.1%。

　　（四）基础设施条件有效改善

　　综合交通建设成效明显。大理至临沧铁路项目建议书获国家批复，玉溪至临沧高速公路前期工作加快推进，省道319线临翔过境公路基本完工，云凤一级公路和沿边三条（段）、德党至勐堆（段）二级公路加快建设，2265公里建制村通畅工程、95公里县乡道改造工程基本完成。沧源得龙机场累计完成投资8.1亿元，孟定机场和永德、凤庆通用机场建设前期工作有序开展。澜沧江流域小湾、漫湾、糯扎渡库区水运基础设施建设基本完工。工程性缺水问题进一步解决。新启动6件小（一）型水库建设，14件骨干水源工程顺利完工，列入“十二五”规划的147件小（二）型水库除险加固全面完成。新增立项耿马、沧源小农水重点县建设项目，实现八县（区）小农水重点县建设项目全覆盖。全市库塘蓄水达到3.2亿立方米，创历史最高水平。耿马坝引水工程开工建设。建成以“爱心水窖”为主的山区“五小水利”工程2.3万件，水利有效灌溉率提高到42%。新解决18万农村人口饮水安全问题。南汀河孟定城区段等河道治理工程稳步推进。“兴地睦边”工程加快实施，完成中低产田地改造30.6万亩。

　　能源通信保障能力明显增强。加快农村电网、骨干电网和城市配套电网建设，户表改造率达到97%。国家15个农村水电增效扩容改造项目全面实施。新开工水电装机4.6万千瓦，新投产水电装机5.4万千瓦。临翔区、云县城市天然气建设项目稳步推进，太阳能光伏发电、风能发电项目前期工作取得新进展。信息基础设施建设加快，4G移动网络覆盖主城区和重点乡镇。

　　（五）城乡建设取得新进展

　　城镇化进程加快。坚持规划引领，积极探索多规合一，启动《临沧市域城镇体系规划》编制，规划建设一批森林城镇。临沧市“十佳建筑”评选工作顺利启动。统筹旧城改造和城市新区建设，一批城市综合体建设加快推进，4600套（户）保障性住房、棚户区改造住房建设顺利开工，1022套临沧主城区改善性住房建设顺利实施。全市完成旧城改造100万平方米，城镇建成区面积增加4.4平方公里。11个特色小镇“一水两污”项目建设加快推进，全市完成城镇污水处理厂配套管网建设55.3公里。建筑业健康发展，增加值增长17.4%。积极推进产城融合，深化户籍制度改革，有序转移农业人口5万人，城镇化率提高到36%。

　　美丽家园建设深入推进。坚持城乡、产业、生态、文化四位一体，不断深化美丽家园建设相关改革，以“硬化、亮化、美化、绿化”为切入点，以产业发展和活动场所建设为抓手，实现房、村、镇、城同步提升。20个传统村落保护工程全面启动，提升美丽村庄110个，改造旧村400个村（组）、危旧房4万户。实现4个建制村、150个自然村整村推进，21个“千户村”启动建设。评选出首批十佳“最美乡村”，“村美、民富、人欢乐”的景象进一步呈现。

　　（六）生态文明建设不断深入

　　生态环境明显改善。始终把生态文明建设放在突出位置。“森林临沧” 、国家可持续发展实验区建设深入推进。从严管控自然保护区、生态功能区、古树名木保护区、水源林保护区，保证森林资源安全，保护生物多样性。持续推进“十百千万”工程，森林城镇、森林村庄建设加快，完成城乡植树559.9万株。临沧中心城市森林公园基本建成，花果山森林公园被评为省级生态文明教育基地。生态综合治理进展顺利，治理水土流失267.5平方公里，完成退耕还林及荒山造林124.2万亩，森林覆盖率提高到66.5%。空气质量优良率100%。

　　环境治理取得新成效。引导全民增强环境保护意识，加强城乡环境保护和环境治理。“采矿、公路建设、生态保护、治污工程建设、工业污染深化治理、农村环境”六大整治行动取得明显成效，矿山、冶炼等重点行业环境安全监管进一步加强，重点减排项目顺利实施。项目环评和环保“三同时”执行率达100%，全市环境质量明显提升。

　　（七）沿边开发开放加快推进

　　边合区建设加快推进。贯彻落实国家沿边开发开放规划和省政府支持临沧边合区建设25条政策。清水河至缅甸登尼二级公路建成投入使用。边合区建设、改革全面启动，托管工作有序推进。沿边金融综合改革试验区建设加快，人民币跨境结算、提高携带人民币现钞进出境限额和开展外币代兑业务等方面取得新突破。口岸基础设施不断完善，通关便利化水平进一步提高。清水河至缅甸腊戍、清水河至缅甸果敢、南伞至缅甸果敢三条边境旅游线路获批开通。边合区入驻企业达149户，一批基础设施、产业发展、民生改善项目加快实施。完成规模以上固定资产投资152亿元，增长43.8%；外贸进出口总额41.1亿元，增长29%。

　　对内对外合作不断扩大。缅北农业合作开发顺利实施，建成产业基地114.4万亩。与斯里兰卡加姆波勒市、俄罗斯杜布纳市交流合作进一步推进。在北京、上海、广州、日本等地开展招商推介活动，积极参加“第2届中国—南亚博览会暨第22届昆交会”、“第十届泛珠会”等活动，成功举办“中华茶商临沧行”、“第四届临沧边境经济贸易交易会”等活动。全市引进市外到位资金668亿元，增长31%，其中：引进省外到位资金511.7亿元，增长38.2%。

　　（八）财税金融平稳运行

　　财政保障能力持续提升。深化财税体制改革，积极培育财源，加强税收征管，建立年初全口径预算编制体系，完成“营改增”试点工作，实现政府预算全面公开。一般公共预算支出完成194.4亿元，增长7.2%。优化预算支出结构，着力保障重大项目、重点工程的实施，重点支持“三农”、教育、卫生、社会保障和就业等民生领域发展，民生支出占一般公共预算支出78.3%。

　　金融支持作用明显增强。加强银政银企合作，信贷投放和融资规模平稳增长。市内银行业金融机构人民币存款余额445.5亿元，增长10.1%；新增贷款56.2亿元，贷款余额377.3亿元，增长17.5%。积极推动“三农”金融改革创新试点，涉农贷款余额208.3亿元，增长22.4%。扎实推进中小微企业融资便利化行动，小微企业贷款余额112亿元，增长36.7%。新型金融机构建设稳步推进，现代保险服务业健康发展。

　　（九）民生持续改善

　　抗震救灾取得阶段性成果。全力抓好“10·7”地震及5.8级、5.9级强余震抗震救灾和灾后恢复重建工作。恢复重建规划全面推进，5697户拆除重建加快推进，5713户受损住房修缮基本完成，受损道路、桥梁、水利、电力、学校、卫生等基础设施修复加固顺利推进，灾区人心安定、社会稳定。

　　就业和社会保障水平稳步提升。强化高校毕业生、城镇困难人员、失业人员的就业服务工作，认真实施《临沧市“新增十万产业大军、推动临沧绿色崛起”人才培养规划》，城镇新增就业2.1万人，农村新增转移就业20.4万人。稳步提高医疗、养老保险覆盖面和待遇水平，城乡居民实行统一的基本养老保险制度，各类保险参保人数达193万人，净增扩面2.6万人，新农合参合率达99.19%。住房公积金保障能力不断提高。城乡低保保障人数达46.2万人，新增养老床位1100张。

　　教育卫生文体事业全面发展。10件惠民实事全部办结。普通高考一本文理综合上线率居全省第2位，临沧师专转型升本取得重大突破，名师、名校工程和校长培养行动计划启动实施，农村义务教育阶段学生营养改善计划和家庭经济困难寄宿生生活补助实现“全覆盖”。以县级公立医院改革为重点的医药卫生改革持续推进。建成标准化乡镇卫生院56个，新建成标准化村卫生室68个，新增住院病床1100张。人口和计划生育、疾病预防控制工作取得新成效。文化体制改革稳步推进，文化惠民措施有效落实。成功举办“永德杯”全国女子柔道锦标赛，在全省第十四届运动会和全省第十届民族运动会、残疾人运动会上取得好成绩，全民健身活动普遍开展。创新型临沧建设加快，科技进步对经济增长的贡献率提高到50.4%。广播电视“村村通”、“户户通”工程完成36.8万户，覆盖率达98%以上。人口较少民族发展扶持力度加大，电站库区万人移民、扶贫整乡整村推进、兴边富民工程顺利推进，农村贫困人口减少12万人。

　　和谐社会建设深入推进。民族团结进步边疆繁荣稳定示范区建设加快。党的宗教政策有效落实，民族和睦，宗教和顺。推动国防建设和经济建设良性互动，荣获新一轮“全省双拥模范城”称号，人民防空综合防护体系建设扎实推进。第三轮禁毒防艾人民战争深入开展，社会治安综合治理全面加强。安全生产“党政同责、一岗双责”有效落实，食品药品监管不断加强。支持工会、共青团、妇联、科协、工商联、关工委、妇儿工委、红十字会、文联、社科联开展工作。侨务、工商、质监、档案、统计调查、地方志工作取得新成绩，老龄、残疾人事业加快发展，防震减灾、气象水文服务能力进一步提高。

　　（十）政府自身建设不断加强

　　民主法治建设扎实推进。自觉接受市人大及其常委会监督，依法执行市人大及其常委会的决定决议，严肃认真地向市人大及其常委会报告工作。支持市政协履行政治协商、民主监督、参政议政职能。积极支持人大代表、政协委员开展视察和调研工作。认真办理市人大代表批评、建议和意见144件，市政协委员提案124件，办复率均达100%。法律“六进”、法治走边关等活动深入开展，“六五”普法和“四五”依法治市工作实现创新发展。

　　服务能力明显提高。政府机构改革、事业单位分类改革、行政审批制度改革有序推进，取消市级部门行政审批事项30项，下放管理层级86项。全面推行五级联动，群众诉求表达渠道更加畅通，信访秩序更加规范。四级便民服务机构标准化建设加快推进，市、县（区）289个部门进驻政务服务中心，企业和群众办事更加方便。推动公共资源项目进场集中交易，完成公共资源交易1656项，各级政务服务中心群众办件办结率达99.9%，政务咨询群众满意率达100%。

　　工作作风明显转变。扎实开展党的群众路线教育实践活动，切实解决“四风”问题。建立健全了学习、调研、服务基层、督查督办、科学民主决策等16项制度，会议、发文数量分别下降31.5%和23.8%。开展23项专项整治，公务用车标识张贴工作全面完成，因公出国境团组个数、人次数、经费支出大幅下降，“三公”经费支出下降12.8%。清理腾退各级党政机关办公用房9533平方米。惩治和预防腐败体系建设进一步完善，监察、审计职能充分发挥，民生资金电子网络监察管理取得良好效果。

　　各位代表，过去一年经济社会发展取得的成绩，是省委、省政府和市委正确领导的结果，是全市各级干部和广大人民群众凝心聚力、开拓进取的结果，是人大代表和政协委员监督、支持、帮助的结果，是社会各界和衷共济、通力协作的结果。在此，我谨代表市人民政府，向广大干部群众、驻临部队、武警官兵，中央、省驻临单位和各类驻临机构，各民主党派、工商联、无党派人士、各人民团体、社会各界人士，向港澳台同胞、海外侨胞和国际友好人士，向所有关心支持临沧发展的海内外人士，表示衷心的感谢并致以崇高的敬意！

　　2014年，全市各级干部和广大人民群众付出了艰辛努力，我市大多数经济指标增幅居全省前列，但生产总值、一般公共预算收入、城镇常住居民人均可支配收入三项指标增速仍低于年初确定的目标。在未来的发展中，我们还面临着一系列困难和问题：经济总量小，扩总量、提质量、转方式的任务仍然很重；基础设施建设滞后，交通瓶颈制约突出；有边不活，沿边开发开放的层次和水平不高；财政公共服务能力不强，投融资难度仍然较大；人才对经济发展的支撑作用不强，创新能力仍然不足；少数干部担当意识、服务意识不强，工作作风有待转变，依法行政的能力有待提升。我们将面对困难，解决困难，开拓创新，奋力前行，全力做好政府各项工作，决不辜负全市人民的重托和期望。

　　二、2015年工作安排

　　2015年是全面深化改革的关键之年，是全面推进依法治市的开局之年，也是全面完成“十二五”规划的收官之年。今年政府工作的总体要求是：全面贯彻落实党的十八大、十八届三中、四中全会、中央经济工作会议精神，以邓小平理论、“三个代表”重要思想、科学发展观为指导，深入学习贯彻习近平总书记系列重要讲话和对云南工作的重要指示精神，按照省委九届八次、九次全会、省委经济工作会议和市委三届七次全会的要求，找准位置，主动作为，紧紧围绕把云南建设成民族团结进步示范区、生态文明建设排头兵、面向南亚东南亚辐射中心，闯路子，走前列。以“构筑生态高地、壮大绿色产业、成就大美临沧”为目标，以加快经济发展，推动产业转型升级为核心，以保障和改善民生为根本，以“三线三区三化”发展为重点，强力推进铁路和高速公路为主的基础设施建设，强力推进沿边对外开放，着力打造“微笑之城、创业之城、森林之城、洁净之城”四城一体的美丽边城，圆满完成今年和“十二五”规划目标任务。

　　今年，经济社会发展的主要预期目标建议为：

　　——生产总值增长11%左右；

　　——规模以上固定资产投资增长25%以上；

　　——规模以上工业增加值增长15%以上；

　　——一般公共预算收入增长10%以上；

　　——一般公共预算支出增长13%以上；

　　——外贸进出口总额增长20%以上；

　　——招商引资实际到位资金增长15%以上；

　　——城镇常住居民人均可支配收入增长12%以上；

　　——农村常住居民人均可支配收入增长14%以上；

　　——社会消费品零售总额增长14%以上；

　　——城镇登记失业率控制在4.3%以内；

　　——单位生产总值能耗下降0.7%；

　　——居民消费价格总水平涨幅控制在3.5%以内；

　　——人口自然增长率控制在7‰以内。

　　11%左右的经济增长预期目标，既立足临沧发展基础，又从同步实现小康目标需要出发，也充分考虑了临沧发展的潜力和可能。认识、适应、引领新常态，前提和关键是依据经济发展规律，把临沧置于全国、全省发展的大格局中，深刻认识临沧在新常态下的地域性、阶段性和个性特征。临沧作为欠发达地区，具有明显的后发优势，从人均生产总值看，开始进入快速发展阶段。继续保持较快的经济发展速度是全国全省区域性结构调整的重要内容，体现了中央、省委关于在新常态下实现经济平稳健康发展的要求，是全市人民的强烈愿望，是我们的责任。

　　临沧困难在边，优势在边，潜力在边，出路在边。加快临沧发展，必须充分彰显沿边优势，努力融入国家“一带一路”和孟中印缅经济走廊建设规划，加快与以缅甸为重点的周边国家的互联互通建设，力争在政策沟通、道路联通、贸易畅通、货币流通、民心相通上取得重大突破；必须加快路网、航空网、能源网、水网、互联网“五网”建设，强力推进高速公路、铁路大通道建设，尽快破解沿边开放交通瓶颈制约；必须全面推进边合区建设，全力打造沿边开放新高地、招商引资和吸引外资的新洼地，加快建设全市经济发展新的增长极，努力实现以开放促开发。

　　做好今年各项工作，我们面临的发展机遇和有利条件主要有：一是习近平总书记视察云南重要讲话，以及李纪恒书记调研临沧重要讲话，为我们指了路、提了神、给了力。二是新一轮西部大开发、集中连片特困地区开发，我市在基础设施建设、产业发展、民生改善等方面将得到国家更多的政策支持。三是我们长期积累形成了良好的发展基础，在产业转型升级、新型城镇化进程中蕴藏着广阔的发展空间和巨大的内需潜力。四是 “生态立市，绿色崛起”发展理念蕴藏着巨大的发展能量。五是随着全面改革、扩大开放的推进，进一步激发了加快发展的活力和动力。六是依法治市的深入推进，将提供有力的法治保障。

　　今年，市政府将着力抓好以下十个方面的工作：

　　（一）着力铁路高速公路建设，加快改善基础设施条件

　　加快综合交通建设。按照最新确定的选线方案，千方百计加快工作进度，开工建设大理至临沧铁路和玉溪至临沧高速公路。加快推进临沧至清水河铁路、临沧至普洱铁路、凤庆至云县至临翔至双江轻轨建设前期工作。抓好大理至临沧、临翔至孟定高速公路建设前期工作。全力推进云凤一体化发展，加快耿马至孟定、临翔至博尚快捷通道建设。扎实推进沿边三条（段）、德党至勐堆（段）二级公路建设。全面完成103公里县乡道改造续建和1227公里建制村通畅工程。新开工1400公里建制村通畅工程和50公里县乡道改造项目。加快改扩建临沧机场，力争沧源得龙机场建成通航，抓好孟定机场和永德、凤庆通用机场建设前期工作。做好澜沧江—湄公河国际航道向上游延伸及临沧港规划建设工作。

　　加快水利工程建设。加快在建的9件中型、20件小（一）型水源工程建设进度，力争开工建设临翔龙浩坝、镇康金场坝、耿马芒枕水库，确保完成3.3亿立方米的蓄水任务。加快推进耿马灌区规划建设，实施永德忙海水库至班卡忙中烟草灌溉连通工程，建好一批沟渠配套项目，抓好云县岔河、耿马允景、镇康帮东河、临翔西河、沧源芒回水库等一批骨干水源工程前期工作。完成2万件以上以“爱心水窖”为重点的山区“五小水利”工程建设，不断提高有效灌溉率，解决农村饮水安全14.2万人以上。加快推进南汀河孟定城区段、幸福段等河道治理工程。继续实施好“兴地睦边”等工程，完成中低产田地改造25万亩。

　　加快能源信息建设。继续加快电网设施建设，确保全市骨干电网、城市配网建设取得新进展，与全省同步实现第一轮农网改造全覆盖。规范发展水电产业，力争新增地方水电装机容量22万千瓦。力争临翔区、云县城市天然气项目建成通气，启动其他县城天然气建设项目。加快太阳能光伏发电、风力发电项目前期工作。完成气象多普勒雷达建设，提高气象服务信息化水平。加快宽带网络普及提速、三网融合和4G业务发展，提高网络性能和信息安全，建设“智慧临沧”。

　　（二）着力沿边开发开放，加快推进对内对外合作

　　突出抓好边合区建设。认真落实好省政府支持临沧边合区建设25条政策，进一步解放思想，大胆探索，力争边合区在开发、管理体制机制和政策上取得新突破。促进基础设施互联互通，全力推进通关便利化，争取清水河口岸尽快与缅方实现对等开放和持护照通行，推动南伞口岸、永和口岸尽快升格为一类口岸，提高沿边金融服务水平，努力实现人员、物资、货币流动流通顺畅，促进边境贸易转型升级。加快推进清水河口岸改扩建等一批基础设施建设，引进更多的企业和项目落户边合区发展。

　　注重提高招商实效。牢固树立发展的希望在民间，活力在民营，动力在民资的理念。创新招商方式，健全完善招商引资工作机制和制度，倾力打造服务最优、门槛最低、审批最简、信用最好、安全最高和低物流、低税费、低要素、低物业、低融通的“五最五低”招商环境。充分利用好南博会、昆交会、泛珠会、2015年北京国际茶业展、第五届边交会等平台，积极走出去和请进来，新引进一批央企、国企、民企落户临沧。切实提高签约项目的履约率、资金到位率和建成投产率。确保新签约项目开工率50%以上，引进市外到位资金768亿元，增长15%以上，其中，省外到位资金589亿元，增长15%以上。

　　积极推进对外交流合作。扩大、深化与缅甸的交流合作，争取与缅甸商务部门尽快建立商务会商机制，进一步建立健全警务、卫生等方面的合作机制，扎实推进缅北农业合作开发，继续推动临沧与腊戍、镇康与果敢尽早结为友好城市，力争与缅甸在经贸、交通、旅游、教育、文化等领域的合作取得新突破。继续深化与俄罗斯杜布纳市、斯里兰卡加姆波勒市友城交流与合作，争取与印度、孟加拉等南亚国家的交流与合作取得新进展。拓展与泛珠三角、长三角、环渤海、京津冀和港澳台等合作，构建全方位对外开放格局。

　　（三）着力新型农村发展综合体建设，加快发展农村经济

　　推进生态文化产业园建设。以现代农业庄园为载体、产业为核心、美丽家园建设为抓手，坚持一个园区突出一个主题，大力实施生态建设、产业提升、村庄打造、文化挖掘和保护、特色旅游开发、品牌创建、素质提升工程，加快建设产业发达、文化富集、丰富多样、景美民富的生态文化产业园。每县至少重点建设1个生态文化产业示范园，带动“龙头企业+产业+农民”的新型农村发展综合体建设，推进三产融合式发展，促进农民增收。

　　提升农业特色产业。推进农业转型升级，加快特色农业向专业化、规模化、集约化、品牌化发展。决不放松粮食生产，确保总产量达103万吨。启动澜沧江流域综合开发规划编制工作，加快四大电站库区建设开发。突出抓好3个省级高原特色农业示范县和现代农业示范园区建设，巩固提升蔗糖、茶叶、核桃、橡胶产业，加快发展澳洲坚果、烤烟、咖啡、木竹、优质肉牛、高原淡水渔业。确保畜牧业产值、渔业产值分别增长5%、20%，农业增加值增长6.5%以上。抓好“三万三带”建设，新建“万元山”30万亩，建设“万元田”10万亩，新培育“万元人”10万人。建设南汀河流域苗木产业带50万亩，建成3个林产品交易中心，打造临翔至清水河林荫大道、林荫河道。

　　创新农业经营体制。坚守耕地红线，加强基本农田保护和耕地质量建设。全面开展农村土地确权发证工作，引导和规范土地经营权有序流转，坚决防止“非农化”现象。积极引导农民采取租赁、入股、抵押、合作等有效形式发展农业产业化，加快转变农业发展方式，千方百计增加农民收入。新增农业产业化组织100个。加快推进打叶复烤、肉牛加工、咖啡加工等项目，新增农业产业化龙头企业 10 户，培育规模以上农业产业化龙头企业5户，实现增加值增长15%。积极推进优质农产品品牌创建，打造一批高原特色农业精品，提高农业综合效益。

　　（四）着力工业园区建设，加快发展绿色工业

　　突出工业园区建设。充分发挥工业园区产业集聚作用，打造区域产业发展新优势。加快推进滇中产业聚集区（新区）临沧产业园规划落地，充分利用滇中地区区位、交通、产业配套和管理优势，加快吸引企业入园发展，支持新材料新能源加工、农产品精深加工、矿产资源精深加工、装备制造五大产业。积极发展市场和资源两头在外的外向型工业。加快实施临沧工业园区“东扩、南移、北进”战略，着力推进临沧通用航空产业园、科技创新园、鑫圆锗业搬迁改造等一批重点项目。突出特色，加快县域百亿元工业园区建设。

　　扎实推进工业项目。继续解决好企业项目申报难、土地落实难、银行贷款难等问题，做好重点项目挂钩帮扶工作，增强煤电油运等要素的综合协调保障，确保规模以上项目竣工19个、新开工项目14个，加快推进在建项目12个、前期工作项目15个，完成规模以上工业固定资产投资184.1亿元，增长25%。确保全社会用电量23.4亿度，增长10%。

　　注重培育各类企业。认真落实好扶持工业发展政策措施，坚持挂钩服务重点企业制度。突出实施好百亿元企业培育工程，抓好上市企业培育、中小企业成长工程和“两个10万元”微型企业培育工作，增强企业实力。促进各类所有制经济共同发展，力争民营经济增加值增长15%以上。选准工业创新驱动主攻方向和突破口，增强企业自主创新能力，打造一批企业品牌、产品品牌。完善能耗水平的监测分析和预警机制，实施好重点节能减排项目。

　　（五）着力旅游产业发展，加快提升第三产业

　　加快生态旅游产业发展。围绕将休闲养生和生态文化旅游业培育成支柱产业的目标，按照“北看茶马，南看阿佤，遍地风光，养生度假”的布局定位，以建设沧源国际旅游度假区为龙头，加快推进澜沧江生态旅游区、百里边关旅游区、佤文化旅游圈、茶文化旅游圈的建设，不断提升全市景区景点的规模和档次。以旅游小镇、恒春小镇、民族特色旅游村建设为抓手，推出一批民族文化、茶文化、影视文化、观光农业、航空观光等多种类型的旅游产品。加大旅游宣传、推荐、营销力度，引进较大旅游企业入驻，办好第三届亚微节、摸你黑狂欢节等重大活动。以假日游、自驾游、跨境游为突破口，实现游客量的快速增长。力争接待国内外旅游者810万人次，增长35%；旅游总收入达59亿元，增长40%。

　　努力壮大服务业。推进服务业提升、融合、创新、跨越，充分释放潜力和活力。坚持传统性服务业和现代服务业同步推进，加快培育以物流、金融、信息、科技、商务服务、养生养老、康体休闲等为重点的生产性服务业和生活性服务业。支持发展连锁经营、物流快递、电子商务等新型流通方式，培育发展限额以上批零住餐企业。加快改善农村市场流通环境，重点建设一批乡镇农贸市场、乡镇基层供销社、区域配送中心，不断完善农村商贸流通网络。力争实现第三产业增加值138亿元，增长10%。

　　（六）着力新型城镇化建设，加快城乡协调发展

　　全面实施“洁净临沧”行动。突出城乡环境卫生综合整治重点，以洁净家园、洁净田园、洁净水源、洁净河流、洁净道路为抓手，努力建设洁净之城。创建100个洁净住宅小区、200所洁净学校、300个洁净单位、400个洁净村庄、5000户洁净庭院示范家园。确保规模化畜禽养殖废弃物和农作物秸秆综合利用率达50％以上，八县（区）城和孟定镇污水处理率达85%以上，居民生活饮用水水质卫生合格率达100%，50%以上河段达到三类水质以上，国、省道公路路面保洁率达80%以上。全面加快城镇建设。以人的城镇化为核心，加快特色城镇化建设。加快推进临沧主城区一城两片、云凤、耿孟“三个一体化”发展。因地制宜推进城镇上山，盘活城镇低效用地和存量建设用地。坚持先规划、后建设，先地下、后地上，实施好城市综合体建设，新开工城镇保障性安居工程9300套（户）、建成10000套（户）以上，全面完成棚户区改造任务，加快推进临沧城区改善性住房建设，规划建设一批城镇停车场、公共厕所等配套基础设施。完成旧城改造100万平方米，新增城镇建成区面积4平方公里。加强城市标准化管理，提高城镇管理服务的科学化、精细化和人性化水平。推动建筑业持续健康发展，确保建筑业增加值增长18%以上。稳步推进撤县设区、撤乡设镇工作。继续深化户籍制度改革，推进城镇基本公共服务向常住人口全覆盖，转移农业人口2.4万人，城镇化率达38%。

　　全面推进美丽家园建设。围绕“村美、民富、人欢乐”的目标，推进集特色农业、生态环境、民族文化和美丽乡村为一体的新农村建设。不断完善农村生产生活基础设施，改善农村人居环境，推进“物的新农村”和“人的新农村”建设齐头并进。认真实施地震安居等工程，加快农村危房改造，新启动一批传统村落保护项目。全力实施连片扶贫开发整乡推进与美丽家园建设、特色旅游小镇打造相结合的“三合一”工程，建设美丽村庄100个，实施农村危房改造4万户。

　　（七）着力森林临沧建设，提高生态文明水平

　　加强生态资源保护。严格执行生态红线管控制度，划定城镇区、耕地保护区、生态保护区、旅游休闲区等红线。尊重自然，充分发挥生态的自我修复能力。强化科学规范管理，建立健全以自然保护区、国家公园、森林公园、保护小区为重点的生态保护体系，落实古树名木挂牌保护制度，保护好森林生态系统、湿地生态系统和珍稀濒危物种资源，切实加强生物多样性保护。

　　强化生态环境治理。持续抓好“十百千万”工程，完成城乡植树575万株。积极创建国家森林城市，推进一批森林乡镇、森林社区、森林机关、森林学校创建，使森林乡镇、森林村庄建成率达40%以上。突出抓好南汀河流域、澜沧江沿线、边境一线生态产业化建设，加快推进退耕还林、防护林建设、石漠化治理、陡坡耕地生态治理、河道治理和农村能源等重要生态建设工程，完成荒山荒地绿化造林2300万株，治理水土流失270平方公里，力争森林覆盖率达67.5%。严格环境执法，加强环境监管，强化环评服务。

　　（八）着力财税金融工作，增强资金保障能力

　　增强财政公共服务能力。坚持为民理财、服务发展，全力争取中央、省财政转移支付补助，强化财源建设，加强收入征管，确保财政收入持续稳定增长。优化支出结构，积极化解政府性债务。加大对民生和重点项目的保障力度，提高财政资金的使用效益，确保民生支出占一般公共预算支出75%以上。创新城投、水投、工投和边投公司等投融资机制，不断扩大投融资规模，缓解资金供求矛盾。

　　提升金融服务发展水平。推进银政银企合作向更深层次更广领域发展，增强企业与金融机构合作成效，努力扩大融资规模，确保市内银行业金融机构新增贷款70亿元以上。支持发展商业类保险，积极开展政策性保险。加快金融组织体系建设，积极规范发展各类融资担保公司、小额贷款公司、资本管理公司、股权投资基金公司，力争新批准3家资本管理公司、增加7家小额贷款公司，加快在临金融机构升格或设立分支机构。加强信用体系建设，改善金融生态环境。

　　（九）着力三大改革，不断深化各项改革

　　抓好三大改革。加快推进生态文明建设相关改革，健全自然资源资产产权制度和用途管制制度，实行最严格的水资源管理和水环境保护制度，探索建立生态补偿机制和资源有偿使用制度，创新生态环境保护管理体制。加快推进边合区建设相关改革，创新沿边投融资体制机制，抓实托管工作，完善边合区实体化管理运行机制，探索建立边合区与八县（区）联动发展机制。推进对缅合作，探索通关互认机制，实施“一站式” 和“三个一”快速通关模式。加快推进美丽家园建设相关改革，深化农村综合改革，创新农垦、供销社、水务等体制机制，完善城市反哺农村工作机制，加快农村人口就地城镇化，推进美丽家园标准体系建设，不断健全城乡发展一体化体制机制。

　　统筹推进各项改革。突出经济体制改革，深入推进行政审批制度、市场监管、财税、金融、土地、小微企业扶持等重点领域关键环节的改革，最大限度地发挥市场在资源配置中的决定性作用。推进社会体制改革，改进社会治理方式，激发社会组织活力。创新有效预防和化解社会矛盾体制，改革信访工作制度，切实提高信访法治化水平。加快就业、社会保障、社会救助、养老服务体系建设，健全社会福利制度。深入推进教育领域综合改革，建立健全教育管理体制，均衡配置教育资源。深化文化体制改革，推进政府由办文化向管文化转变。继续深化县级公立医院综合改革，统筹推进医疗保障、医疗服务、公共卫生、药品供应、监管体制综合改革，提高城乡医疗卫生服务能力。

　　（十）着力社会事业发展，不断保障和改善民生

　　抓好震后恢复重建。坚决贯彻落实中央、省对“10·7”地震及5.8级、5.9级强余震恢复重建的部署，优先解决好受灾群众最关心最迫切的住房、学校、医院等问题。加快受损民房恢复重建，力争拆除重建农户春节前入住新居。加快教育、卫生等公共服务设施和水利、交通等基础设施的恢复重建，确保年底前基本完成恢复重建任务。

　　加快发展教育卫生科技文体事业。坚持教育优先发展，推进普通高中、职业教育、特殊教育、学前教育和民办教育均衡发展。加强省级高级完全中学建设，加快推进临沧师专转型升本，继续巩固农村义务教育阶段学生营养改善计划和家庭经济困难寄宿生生活补助“全覆盖”成果。加强医疗卫生服务体系建设，健全卫生人才队伍建设长效机制，确保市人民医院新院投入使用，加快推进标准化乡镇卫生院和村卫生室建设。坚持计划生育基本国策，全面做好人口工作，努力提高人口素质。抓好新一轮创新型云南行动计划的实施，强化企业技术创新主体地位，加快国家可持续发展实验区建设。加大文化惠民工程实施力度，加强物质和非物质文化遗产保护，继续实施广播电视“户户通”等工程，扩大市、县（区）本地节目覆盖面。广泛开展全民健身活动，认真做好全省第十五届运动会、全省第十一届民族运动会、残疾人运动会和全省第九届农民运动会在临沧举办的筹备工作。

　　做好劳动就业社会保障工作。坚持就业优先，确保城镇新增就业2.5万人，农村劳动力就业培训15万人。增强社会保障，确保各类保险参保人数达193万人以上，确保新农合参合率继续保持在98%以上。建立机关事业单位养老保险制度，鼓励企业建立商业养老健康保障，建立健全以经济困难、高龄、失能老年人为重点的老年福利制度，鼓励民资、外资兴办营利性养老机构。完善城乡低保、农村五保供养、医疗救助、双拥优抚政策，全面落实临时救助，关爱农村“三留守”群体。积极发挥住房公积金互助作用，支持职工改善住房条件。

　　全面推进和谐临沧建设。加快推进民族团结进步边疆繁荣稳定示范区建设。认真抓好宗教领域的团结稳定工作，促进宗教和谐。重视扶持人口较少民族发展，决不让一个兄弟民族掉队，不让一个民族地区落伍。深入开展兴边富民行动，坚持区域发展和精准扶贫并举，认真总结推广腾龙经验和南美模式，加快整乡整村等扶贫项目建设，减少农村贫困人口12万人。深入推进第三轮禁毒防艾人民战争，广泛开展双拥共建活动。全面落实“四五”依法治市和“六五”普法规划。深入开展安全生产专项整治，加强食品药品安全目标管理。全面开展平安建设，严密防范、依法打击各种违法犯罪活动，提高群众安全感和满意度。加强边境处突维稳能力建设，切实维护好边境的稳定和安全。支持工会、共青团、妇联、科协、工商联、社科联、文联、关工委、妇儿工委做好工作。支持红十字会、慈善、残疾人等事业健康发展。提升防震减灾、水文服务能力。加强统计调查、工商、供销、粮食、档案、地方志、侨务、人防、质监等工作。

　　三、切实加强政府自身建设

　　扩大党的群众路线教育实践活动成果，扎实开展“三严三实”和“忠诚干净担当”专题教育，建设职能科学、结构优化、廉洁高效、人民满意的法治政府、责任政府、服务型政府。

　　（一）加强法治政府建设

　　依法履行职能。加快转变政府职能，推进机构、职能、权限、程序、责任法定化。理清政府与市场边界，理顺政府与市场、社会的关系。完善机构编制管理制度和规则，合理划分各级各部门权责。编制和公开政府行政权力清单、责任清单，坚持法定职责必须为、法无授权不可为。依法优化和公开权力运行流程，确保权力在法律制度的框架内运行。完善行政决策程序，落实公众参与、专家论证、风险评估、合法性审查、集体讨论决定。严格执行重大执法决定法制审核制度，建立健全依法行政责任追究制度。

　　强化权力制约和监督。自觉接受市人大及其常委会的法律监督和工作监督，执行市人大及其常委会的决议决定，接受市政协的民主监督，积极采纳市政协的意见建议。认真办理人大代表批评、意见、建议和政协委员提案。虚心听取和采纳民主党派、工商联、无党派人士、各人民团体的意见，接受新闻舆论监督和社会公众监督。加强行政复议监督，建立行政复议与行政诉讼、信访工作联动机制。完善审计监督，落实对公共资金、国有资产、国有资源和领导干部履行经济责任情况实行审计全覆盖规定。深入推进政务决策公开、执行公开、结果公开。

　　公正文明执法。加快综合执法改革，重点加强食品药品安全、公共卫生、安全生产、资源环境、交通运输等重点领域的综合执法。严格落实行政执法人员持证上岗和资格管理制度。完善行政执法程序，重点规范行政许可、行政处罚、行政强制、行政征收、行政收费、行政检查等关系群众切身利益的执法行为。建立健全行政裁量权基准制度，规范裁量范围、种类、幅度。落实行政执法责任制和执法评议考核制，严格确定不同部门及机构、岗位执法人员执法责任和责任追究机制。

　　（二）加强责任政府建设

　　增强担当能力。坚持开拓创新，始终把解放思想、锐意创新作为政府工作的根本动力。不断提高学习能力、判断能力、决策能力、服务能力和人格魅力，敢想、敢试、敢闯，敢于担当，推动发展。多思发展之策，多行务实之举，善于用改革的办法和创新的举措突破瓶颈制约，破解发展难题。

　　增强执行能力。坚持务实高效，始终把求真务实作为政府工作的自觉追求。把更多的心思和精力放在抓落实上，健全重点工作和重大项目任务分解落实机制，严格执行行政问责、首问首办、服务承诺、限时办结等制度，努力提高办事效率。令行禁止，强化督查，坚决消除工作中的“梗阻”现象。

　　切实转变作风。发扬雷厉风行的作风，形成“你追我赶，等不起、慢不得、坐不住”的干事氛围。深入基层，深入群众，倾听群众呼声，切实帮助基层群众解决实际问题。加大治庸、治懒、治散力度，形成用结果说话，重实干、求实效的工作导向，进一步提高行政效率。

　　（三）加强服务型政府建设

　　加强政风建设。严格执行中央“八项规定”，认真落实省委、市委实施办法，坚决反对形式主义、官僚主义、享乐主义、奢靡之风。严格控制会议、庆典、研讨会、论坛的数量和规模，严格控制公款出省、出国（境）考察活动，严格执行公务人员出差、会议、培训等经费管理制度。深怀为民之心，恪守为民之责，善谋为民之策，常行为民之举，全心全意为人民谋利益。

　　加强廉政建设。认真履行“一岗双责”，落实主体责任。深入推进以完善惩治和预防腐败体系为重点的反腐倡廉制度建设，健全重大事项报告制度、质询制度和民主评议制度。严格监管土地批租、工程招标、产权交易等腐败易发多发环节，从源头上防止腐败行为的发生。严肃查处各类违纪违法案件。强化行政监察，认真纠正部门和行业的不正之风，营造风清气正的良好氛围。

　　各位代表！“三阳开泰，前景光明”。让我们紧密团结在以习近平同志为核心的党中央周围，在市委的坚强领导下，科学认识经济发展新常态，主动适应经济发展新常态，以只争朝夕、科学求实的态度，抢占先机，彰显优势，提气聚力，扎实工作，努力开创全市经济社会发展新局面，奋力谱写好中国梦临沧篇章！
